# Artificial Neural Networks for the Diagnosis of Aggressive Periodontitis Trained by Immunologic Parameters

**DOI:** 10.1371/journal.pone.0089757

**Published:** 2014-03-06

**Authors:** Georgios Papantonopoulos, Keiso Takahashi, Tasos Bountis, Bruno G. Loos

**Affiliations:** 1 Center for Research and Applications of Nonlinear Systems, Department of Mathematics, University of Patras, Patras, Greece; 2 Department of Conservative Dentistry, School of Dentistry, Ohu University, Fukushima, Japan; 3 Laboratory of Nonlinear Systems and Applied Analysis, Department of Mathematics, University of Patras, Patras, Greece; 4 Department of Periodontology, Academic Center for Dentistry Amsterdam (ACTA), University of Amsterdam and VU University, Amsterdam, The Netherlands; University Hospital of the Albert-Ludwigs-University Freiburg, Germany

## Abstract

There is neither a single clinical, microbiological, histopathological or genetic test, nor combinations of them, to discriminate aggressive periodontitis (AgP) from chronic periodontitis (CP) patients. We aimed to estimate probability density functions of clinical and immunologic datasets derived from periodontitis patients and construct artificial neural networks (ANNs) to correctly classify patients into AgP or CP class. The fit of probability distributions on the datasets was tested by the Akaike information criterion (*AIC*). ANNs were trained by cross entropy (*CE*) values estimated between probabilities of showing certain levels of immunologic parameters and a reference mode probability proposed by kernel density estimation (KDE). The weight decay regularization parameter of the ANNs was determined by 10-fold cross-validation. Possible evidence for 2 clusters of patients on cross-sectional and longitudinal bone loss measurements were revealed by KDE. Two to 7 clusters were shown on datasets of CD4/CD8 ratio, CD3, monocyte, eosinophil, neutrophil and lymphocyte counts, IL-1, IL-2, IL-4, INF-γ and TNF-α level from monocytes, antibody levels against A. *actinomycetemcomitans* (*A.a.*) and *P.gingivalis* (*P.g*.). ANNs gave 90%–98% accuracy in classifying patients into either AgP or CP. The best overall prediction was given by an ANN with *CE* of monocyte, eosinophil, neutrophil counts and CD4/CD8 ratio as inputs. ANNs can be powerful in classifying periodontitis patients into AgP or CP, when fed by *CE* values based on KDE. Therefore ANNs can be employed for accurate diagnosis of AgP or CP by using relatively simple and conveniently obtained parameters, like leukocyte counts in peripheral blood. This will allow clinicians to better adapt specific treatment protocols for their AgP and CP patients.

## Introduction

Periodontitis is a bacterial-driven chronic inflammatory destructive disease of the tissues surrounding and supporting the dental root [1]. Severe periodontitis affects around 8.5% of the general population, while a moderate form of the disease is present in 30% and a mild form in 9% of adults aged 30 and older [2].

Periodontitis is a complex disease, where multiple causal factors simultaneously and interactively play a role. There are four main causal risk factors, i.e. the subgingival microbiota (the bacterial biofilm), individual genetic variations, life style and systemic factors [3]. It is a well-known fact that the behavior of a complex system cannot be explained by isolating its components [4]. Currently two clinical types of periodontitis are recognized; the aggressive (AgP) and the chronic (CP) form [5]. Due to the complexity of the pathogenesis of the disease, there is no single clinical, microbiological, histopathological, genetic test or combinations of them to discriminate AgP from CP patients [6].

Clinical identification of AgP cases is based on rapid attachment loss and bone destruction, the absence of systemic factors to explain this progression rate and familial aggregation [7]. Any age upper limit in discriminating AgP from CP is arbitrary. Nevertheless, given the same amount of periodontal destruction individuals with AgP are found considerably younger than CP patients. The age of 35 has been used as a cut-off point to discriminate between AgP and CP [8]. It is realized that is difficult to distinguish between the two phenotypes at the initial stages of periodontitis, thus preventing proper early clinical management of AgP, which is generally found more demanding.

Complexity is understood through modeling and simulation [4]. In a recent study [9] using cellular automata experiments, periodontitis was described as a system out of equilibrium with the level of the host immune response determining its entropy rate. In a subsequent study [10] a chaotic map was analyzed, expressed by a particular equation, which accurately models periodontitis progression in connection to the variation of the host immune response level. By renormalization arguments, two zones of disease activity were identified, a fast and a slow progressing zone, corresponding to AgP and CP respectively. Based on the above, we may now pose the hypothesis that different entropy rates might indeed reflect the presence of distinct patient clusters in immunologic and clinical datasets.

Histograms are the oldest probability density estimators [11], but suffer from certain important drawbacks; they are discontinuous and hardly appropriate for representing bivariate or trivariate data. Nonparametric kernel density estimation (KDE) methods on the other hand, reveal structure in datasets, such as skewness and multimodality that might be missed by classical parametric methods [12]. KDE is an unsupervised learning procedure that can be used for nonparametric classification tasks [13]. In general, when a desired outcome is known, a learning process is called supervised, otherwise it is unsupervised learning.

Artificial neural networks (ANNs) are considered powerful nonlinear statistical tools to model complex relationships between inputs and outputs. Therefore, they appear appropriate in searching for parameters that could achieve an accurate diagnosis of AgP or CP. ANNs consist of a set of simple units called neurons by analogy with the biological neurons [14]. Neurons are linked to each other by a weighted connection which is called synapsis, and are organized in layers: Information is fed to neurons of the input layer, and then processed in the hidden layer and finally exits to the neurons of the output layer. ANNs can be adaptive to external or internal changes and “learn” from the data entered into them. For instance, one type of ANN is the multilayer perceptron (MLP); this is a feedforward ANN trained by the backwards propagation of the error found in the outcome layer. It can be used for supervised learning classification procedures.

The first aim of this study was to estimate the probability density functions of a set of observed clinical and immunologic data in periodontitis patients. Secondly we investigated the fit of the data to various probability distribution models. Based on these findings we developed ANNs able to classify periodontitis patients belonging to either one of the two different clinical forms, aggressive or chronic form.

## Materials and Methods

### Study population

Data were retrieved from previous studies that provided 4 distinct patient samples. From one study [15], we obtained 29 periodontitis patients with severely advanced disease as evidenced by clinical and radiographic examination, which were clinically followed and maintained for 5 to 8 years (we designate this as sample-1). At baseline examination, they had ≥14 teeth present and at least 50% of their teeth showed bone loss of ≥50% of their root length. We used data on baseline radiographic mean bone loss and on longitudinal mean radiographic bone loss level change. From Loos et al. [16], [17] studies from 76 periodontitis patients (the same group for both studies to which we will refer as sample-2) we derived datasets of total number of monocytes, lymphocytes, basophils, neutrophils and eosinophils in peripheral blood, as well as the total number of CD3, CD4, CD19 cells and the CD4/CD8 ratio. For sample-2 radiographic bone loss measurements were also available (% of teeth with bone loss of ≥50% of the tooth root length). From Graswinckel et al. [18] we used the datasets for IgA, IgM and IgG from 80 periodontitis patients (sample-3). From Takahashi et al. [19] (sample-4) we derived data of serum antibody levels in 162 periodontitis patients against *Aggregatibacter actinomycetemcomitans* (*A.a.*) (Y4 antigen), *A.a.* (ATCC 29523), *A.a.* (SUNY67), *Porphyromonas gingivalis* (*P.g.*) (FDC381), *P.g.* (SU63), *Eikenella corrodens* (*E.c.*) (ATCC 23834), *Prevotella intermedia* (*P.i.*) (ATCC 25611), *Prevotella nigrescens* (*P.i.*) (ATCC 33563), *Capnocytophaga ochracea* (*C.o.*) (S3), *Wolinella succinogens* (ATCC 29543), *Treponema Denticola* (*T.d.*) (ATCC 35405) and *Fusobacterium nucleatum* (*F.n.*) (ATCC 25586). In addition we derived data of IL-1, IL-2, IL-4, IL-6, TNF-α and INF-γ levels produced by mononuclear cells from peripheral blood.

The undefined periodontitis patients, those with adult periodontitis (AP) or those with localized (L) or generalized (G) early onset periodontitis (EOP) from the studies from which data were retrieved [16]–[19] were reclassified as previously described [9]. Those with an age at the time of diagnosis >35 years or originally having AP, were reclassified as CP; patients ≤35 years were classified as AgP; those with L- or G-EOP were all classified as AgP. EOP (a term used in the 1989 world workshop in clinical periodontics, preserved in the 1996 modification and changed to AgP in 1999) is considered to have its onset from puberty until 35 years [20].

Therefore from sample-2 we derived 23 AgP and 53 CP cases; from sample-3 18 AgP and 62 CP cases. For these two samples we had an exclusion of 20% of the initially recruited patients for various reasons that could affect their immunologic profile (like chronic medical disorder, pregnancy, trauma, recent tooth extraction, etc). From sample-4 we obtained 68 AgP and 43 CP cases. A group of 51 patients “suspected for EOP”, was declared suspected for AgP with no definitive criteria for a final diagnosis; they had severe periodontitis and a disease history that suggested EOP, but were >35 years at the first examination and with no family members diagnosed with EOP. Patients in sample-4 were recruited as they presented at the Okayama University Dental Hospital over a period of 10 years.

### Kernel density estimation

For the estimation of univariate or bivariate probability densities of the data distribution of the various parameters, an appropriate kernel function is needed [21]. The process of choosing a kernel function is described in Text S1 in File S1.

### Fit of the data distributions to probability models

The fit of the available data distributions was tested in five well known probability models for continuous variables: the Normal, the Exponential, the Weibull, the Pareto and the Gamma models. They all have been extensively used and applied on biological systems [22]. A first visual appreciation of the fit was judged by quantile to quantile (Q-Q) plots. Subsequently, comparisons between models were based on the Akaike information criterion (*AIC*) [23], [24], which safe-guards against overfitted models [25].

### Construction of artificial neural networks

**Figure 1:**
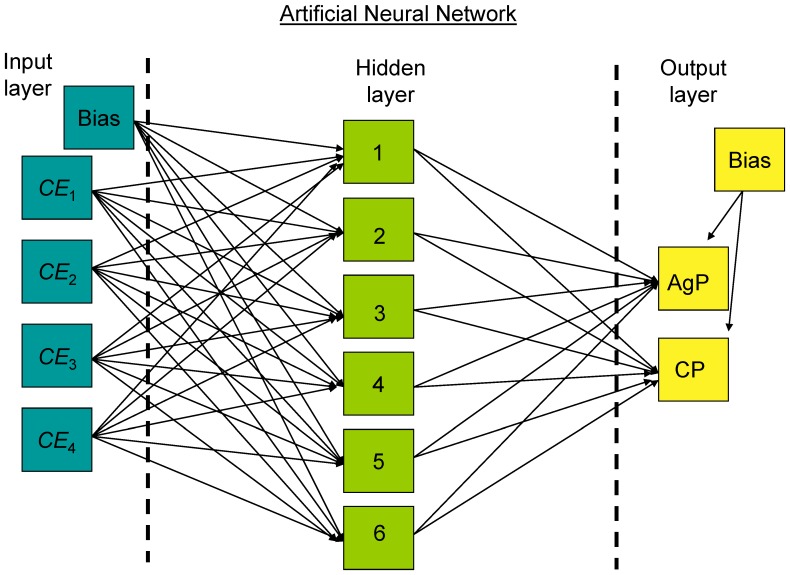
Multilayer perceptron feedforward neural network with error backpropagation. The information (cross entropy values of immunological parameters for each patient) is inserted in the input neurons. At the hidden layer, here with 6 neurons, we sum the information and transfer it (through the sigmoid function) to the outcome layer, where the sigmoid function exits an AgP or CP verdict. Bias neurons have a constant value and help the network to learn patterns. They are independent from other neurons and can shift the curve of the sigmoid function to the left or to the right. The classification error found at the outcome layer backpropagates in the network and synaptic weights are adapted accordingly as the network learns from its error and tries to minimize it.

We built MLP ANNs to classify periodontitis patients. A diagram of the MLP ANN applied in this study is presented in Figure 1. It depicts the three types of layers, the input, the hidden and the output layer along with the interweaving of their neurons. We trained ANNs using cross entropy values (*CE*) [26] of immunological parameters of periodontitis patients in reference to a target probability value revealed by KDE.

The first step in the construction process was to calculate the probability *p*(*x*) of demonstrating a certain level of an immunological parameter (*x*) in an individual patient. We used for that the cumulative probability function (cpf) of the corresponding probability model. At a second step, we computed cross entropy (*CE*) values [26] for each patient between the previously described probability *p*(*x*) for selected immunologic parameters and a reference probability value, the target probability *t_i(CP)_*. We used the formula
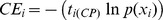
where i = 1, 2, …N and N is the number of the immunological parameters (*x*) inserted into the ANN. *CE* is a nonsymmetric measure of the difference between two probability distributions. The target probability distribution *t_i(CP)_* was estimated by direct application of the appropriate cpf; we used the mode value of *x* with the highest density probability of the immunologic data distribution (indicated by KDE) as the reference point. We assumed that the highest density modes represent clusters inhabited mostly by the CP patients.

Our pruning strategy in feature selection at the input layer was based on automatic relevance determination (ARD) [27]. According to the method, features whose posterior weight distributions show low variance are discarded. The weight decay regularization parameter was determined by a 10-fold cross-validation process [28] (see text S1). If 10-fold cross validation is used for the determination of the weight decay regularization parameter, usually there is no need to use cross validation to determine the number of the hidden units [28]. We determined the number of hidden units and the maximum number of epochs by experimentation (we stopped increasing iterations when the sum of squares error stopped improving) [27], [28]. Finally, since the results of the networks are sensitive to the initial weight values, we tried 10 random initial weight configurations and we computed the mean prediction rates [28]. We report the technical features of the ANNs, such as maximum number of epochs (iterations) and learning methods applied, as well as sensitivity, specificity and overall accuracy of the ANNs against the original clinical diagnosis.

## Results

**Figure 2:**
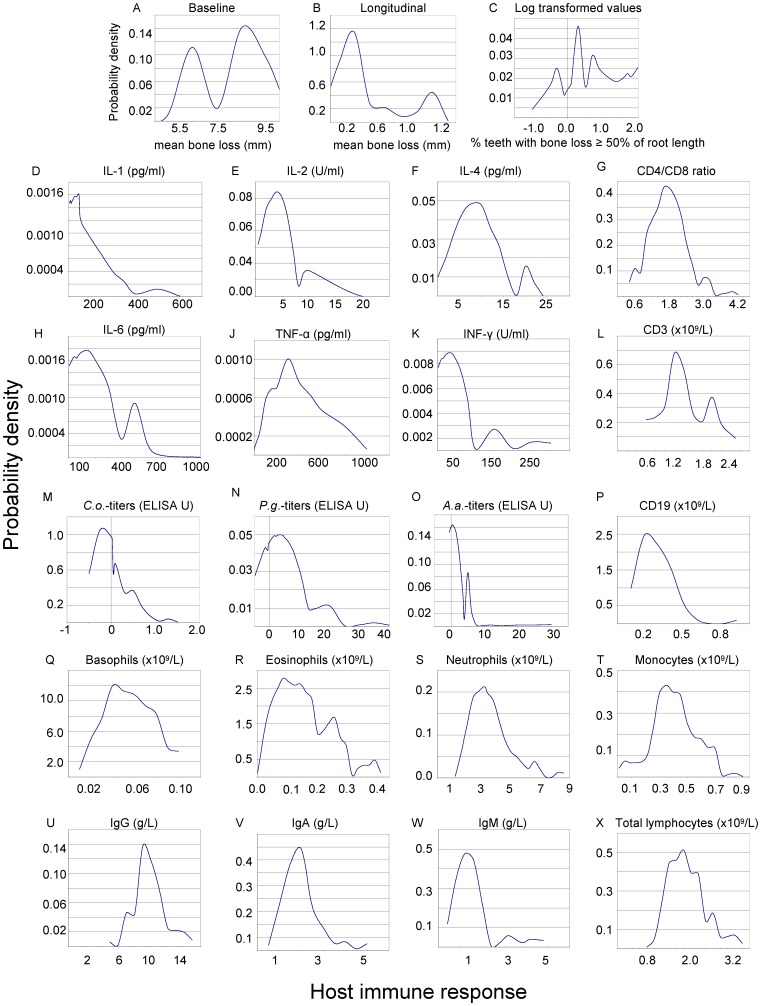
Univariate kernel density estimation (KDE) graphs. Graphs A to C. Univariate KDE for radiographic bone loss measurements: modes (single, bimodal or multimodal) are defined as the values that appear more frequent. Graphs A & B from sample-1. In graph C (sample-2) we log transformed the confined data to find support in the interval (−∞, +∞) (see text S1). Graphs D to X. Univariate KDE for immunologic data: possible evidence of multimodality for the CD4/CD8 ratio, CD3, lymphocytes, monocytes, eosinophils, basophils and neutrophils counts (sample-2), IgG levels (sample-3), IL-2, IL-4, IL-6, INF-γ, TNF-α, IgG *A.a.* titers and IgG *C.o.* titers (sample-4). Mini clusters close to each other are detected for IL-1 and IgG *P.g* titers (sample-4).

From 4 distinct samples we derived clinical and immunologic data and performed KDE. From sample-1 on baseline and longitudinal bone loss data KDE revealed possible evidence of two clusters of patients (Figure 2A, B). On sample-2 for the dataset of % of teeth with bone loss ≥50% of their root length, possible evidence of two main clusters was also found (Figure 2C). KDE on sample-2 revealed three to seven clusters for monocytes, basophils, neutrophils, eosinophils and lymphocytes counts (Figure 2Q, R, S, T & X) and two clusters for CD3 (Figure 2L). From sample-3, IgA and IgM data showed one mode (Figure 2V & W) while IgG data (Figure 2U) showed two clusters. From sample-4, KDE gave possible evidence of two main clusters for IL-2, IL-4, IL-6, TNF-α, INF-γ, (Figure 2D, E, F, H, J & K), and of 2 to 3 modes for antibody IgG titers against the 12 examined bacteria (graphs are shown for three of them) (Figure 2M, N & O).

**Figure 3:**
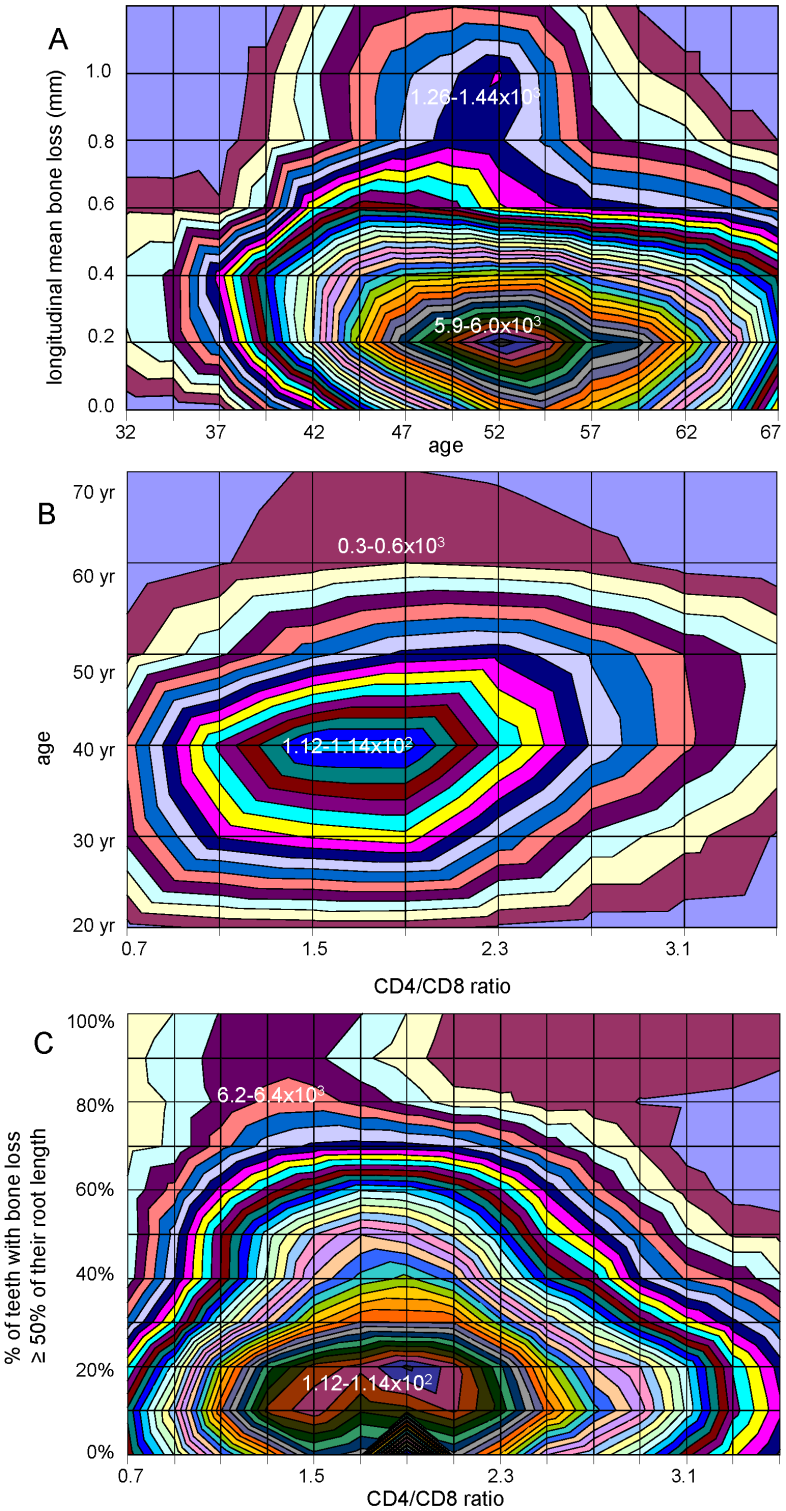
Bivariate kernel density estimation (KDE) for some selected parameters. (A) Contour plot for bivariate KDE of longitudinal radiographic bone loss level (sample-1) in relation to age: this topographical-like plot shows a main cluster with 0.2 mm longitudinal bone loss and a small cluster with almost five times greater bone loss. (B) Contour plot for bivariate KDE: By estimating probability density for CD4/CD8 ratio by age (sample-2), we see two clusters although not separated distinctly, at modes of 1.5 and 1.9. (C) Contour plot for bivariate KDE: By estimating probability density for CD4/CD8 ratio (sample-2) by disease severity (% of teeth with bone loss ≥ of 50% of their root length), we reveal two distinct clusters of patients, with modes at *x* values of 1.5 and 1.9.

Some bivariate KDE were generated. Using longitudinal bone loss data in relation to age (sample-1), we identified two clusters of patients (Figure 3A). The majority of patients clustered around the mode of 0.2 mm of longitudinal bone loss over the follow up period, while a small cluster of patients showed a mode with an almost 5 times higher value for this parameter. In the bivariate KDE of the CD4/CD8 ratio in relation to age (sample-2) (Figure 3B) or in relation to % of teeth with bone loss ≥50% of their root length (Figure 3C), two clusters at modes *x* = 1.5 and *x* = 1.9 are found.

We found the baseline and longitudinal bone loss measurements to fit to the Normal model (Table S1 in File S1). Most of the immunologic data fitted to the Gamma model (Figure S1, Table S1 in File S1). We built three ANNs with three kinds of immunologic parameters as inputs: leukocytes (ANN_1_) (from sample-2), interleukins (ANN_2_) and IgG antibody titers (ANN_3_) (from sample-4). We didn't mix parameters from the two samples. The results of ARD on feature selection are presented in Table S2 in File S1. ANN_1_ showed high accuracy (98.1%) followed close by ANN_2_ (95.6%), while ANN_3_ was left behind in overall accuracy (90%) (Table 1). For comparison to the ANNs performance, we conducted canonical discriminant analysis and binary logistic regression using the above selected inputs. They both displayed inferior results compared to the ANNs (Table S3 in File S1).

**Table 1:** Characteristics of three artificial neural networks (ANN) built on immunological parameters.

Input neurons	Network's description	Results^f^
ANN_1_		
*CE* a values of	#^o^ of hidden layers = 1,	Sensitivity = 98,6%,
1. CD4/CD8^b^	# of neurons in hidden layer = 9,	Specificity = 97.9%
2. Neutrophils	max. # of epochs^c^ = 900,	Accuracy = 98.1%
3. Monocytes	weight decay regularization parameter^d^ = 0.0001,	
4. Eosinophils	learning method = batch^e^, gradient descent.	
ANN_2_		
*CE* values of	# of hidden layers = 1,	Sensitivity = 92.3%,
1. IL-1^g^	# of neurons in hidden layer = 10,	Specificity = 96.9%
2. IL-4	max. # of epochs = 800,	Accuracy = 95.6%
3. IFN-γ^h^	weight decay regularization parameter = 0.0005,	
4. TNF-α^i^	learning method = batch, gradient descent.	
ANN_3_		
*CE* values of	# of hidden layers = 1,	Sensitivity = 91.1%,
1. *A.a.* k titers	# of neurons in hidden layer = 10,	Specificity = 89.4%
2. *P.g.* l titers	max. # of epochs = 1000,	Accuracy = 90.0%
3. *C.o.* m titers	weight decay regularization parameter = 0.005,	
4. *F.n.* n titers	learning method = batch, gradient descent.	

a CE = Cross entropy. Feature selection by automatic relevance determination.

b CD = cluster of differentiation.

c epoch = iteration.

d Determined by 10-fold cross validation.

e Batch training passes all input data before updating the synaptic weights.

f The mean values of 10 random configurations of initial weights are reported (mean values of sensitivity, specificity and overall accuracy of the ANNs against the original clinical diagnosis).

g IL = interleukin.

h INF = interferon.

i TNF = tumor necrosis factor.

k *A.a.* = *Aggregatibacter actinomycetemcomitans*(Y4 antigen).

l *P.g.* = *Porphyromonas gingivalis*(FDC381 antigen).

m *C.o.* = *Capnocytophaga ochracea*.

n *F.n.* = *Fusobacterium nucleatum*.

o # = Number.

## Discussion

We found by KDE techniques possible evidence of two modes in radiographic bone loss and selected immunologic data. We also fitted probability models to our datasets. In training ANNs we used *CE* values instead of original data. This might seem an unnecessary complication, but the ANNs were trained far better providing higher prediction accuracy. We can only speculate on the reason for that. It might have been that the smoothing parameter in KDE fine-tuned the complexity of ANNs via a shrinking effect on weights; increasing complexity directly relates to an increasing variance of ANNs test error and poorer prediction outcome [28], [29]. The nonsymmetric nature of *CE* might also have facilitated the learning process.

ANNs have been used in monitoring medical conditions, where a complex combination of changes in multiple variables is associated with the onset of a disease [30]. ANNs simulate the *tabula rasa* or clean-slate learning we find associated with biological processes [14]. ANNs using the softmax transfer function and the *CE* error function are equivalent to linear logistic regression in the hidden units [28]. However, a growing number of studies in various scientific fields indicate that ANNs provide higher prediction accuracy than multiple regression models in solving classification problems [31]. This was also shown by our results (Table S3 in File S1). We must note however, that the performance of an ANN is variable, depending on the learning method used. In fact, ANNs in supervised machine learning methodology are found to approximate any function with arbitrary accuracy. However, they are susceptible to the overfitting problem [29]. Our results indicate that ANNs can be helpful in the diagnosis of AgP in a periodontal practice, while they appear unsuitable for monitoring the general population where a test with almost 100% specificity is required.

Clustering periodontal patients to gain insight into the pathogenesis of periodontitis is not a new idea. Among the vast literature, we can distinguish a study that analysed differences among 5 groups of patients [32]; grouping was based on pocket depth (PD) and bleeding on probing scores (BOP). Using logistic regression *C. rectus* antibody titers was the best single predictor among all IgG titers of one of the 5 phenotypes and *P. gingivalis* titers found the best single predictor of other three phenotypes. The study supported the microbial specificity of periodontitis pathogenesis. However, the discontinuity of the grouping method used in the study, like in the use of histograms, translates into inefficient use of the data and causes huge difficulties when derivatives of the estimates are required. The situation can be perplexed by the combination of two parameters (PD and BOP). In contrast, KDE when used as intermediate component of another method, like in the current study, is particularly justified as an alternative to histograms.

The main body of the periodontal literature relevant to cluster analysis is based on similarities of subgingival microbiota, followed by investigation of clinical and immunologic differences among clusters. For example, hierarchical cluster analysis identified 5 groups of AgP patients of similar subgingival microbiota [33]; IL-1β/IL-10 ratio in gingival crevicular fluid (GCF) was significantly different among groups. A recent review underscored the fact that although bacteria initiate periodontitis, disease progression is multidimensional and poorly understood [34]. Our unsupervised grouping method on immunologic parameters from peripheral blood determined clusters by local maxima of the overall density function. However, one can question the validity of the *in vitro* interleukin evaluation tests. Using parameters from GCF or saliva [35] is an alternative that may enhance the prediction or generalization ability of ANNs. Our hypothesis that the host immune response level is the determinant of periodontitis disease rate [8], agrees with the recently proposed paradigm for periodontitis pathogenesis [36]; it is suggested that even the immune response level mounted at the early stage of gingivitis is the determinant factor of periodontitis progression and not the presence of specific bacteria known for their virulent properties. On this basis it becomes meaningful to use immunologic parameters by nonlinear methods to discriminate AgP from CP.

It is currently understood that for the behavior of a complex disease many components intricately and dynamically interact; the emergence of the behavior of a complex system cannot be explained by considering its contributing parts separately (the whole does not equal the sum of the parts) [4]. The biological relevance of our results can be evaluated through the above realization. A complex system is not static: it undergoes continuous scale transformations. At one scale some factors compete to each other and at another scale below or above they act in synergy. That makes it difficult to find significant differences between AgP and CP when simple comparisons are made, for example by mean values of immunologic parameters. On the other hand, ANNs start nearly linear (with weights near zero) and become nonlinear as the weights increase [28]. As they grow they learn the nonlinear patterns of the data through the backpropagation of their misclassification error. However, the problem with ANNs remains their generalization ability and overfitting is always a concern [28], [29].

The limitations of the methodology used in this study should be addressed. First of all, there are no rules for determining how large a sample should be for justifying the application of ANNs. However, having larger samples would allow us to leave a portion of the patients entirely out of the training process to finally evaluate upon this portion the general performance of the models. This seems to be an objective way to test the generalization ability of the models. Secondly, regarding the ANNs design, it is better to have too many hidden units than too few. The number of hidden units varies in relation to the number of inputs and the size of the training sample, usually being in the range of 5 to 100 [28]. With too few hidden units, the model might not have enough flexibility to capture the nonlinearities in the data; with too many hidden units the model will suffer from overfitting. Overfitting is avoided by early stopping or regularization. A common technique of regularization is to add a penalty in the error function, as we actually did (see text S1). By early stopping or by regularization we want to stop training well before we approach the global minimum, where neural networks with too many weights generally overfit the data. Third, not removing irrelevant input features would affect the classification accuracy of the network. Feature pruning typically increases the generalization ability of classifiers [29]. It is realized, however, that a variable, even though useless by itself, can be useful in combination with others [37]. Therefore, a limited set of candidate features may miss a potent combination of features. On the other hand, the variance-bias trade-off problem calls for our attention when we increase the complexity of the model. Too many features will decrease the training error by overfitting and at the same time will increase the test (generalization) error [28].

The diagnostic criteria for AgP and CP were different among the 4 patient groups used for this study. One sample [15] was selected on the basis of the presence of severe periodontitis with no discrimination of AgP or CP. The sole criterion of disease severity was obviously inadequate to predict the future behaviour of all patients. A small subgroup of patients showed a 5 times higher rate of longitudinal bone loss than the main cluster of patients (Figure 3A). This is definitely a group of patients with aggressive disease behavior. Takahashi's group [19] used the current classification scheme [5]; however, discrimination of AgP and CP was as much as possible precise, resulting in a group of “suspected” for AgP patients, which contributed to KDE but was not used in ANNs. The other two samples used in this study suffered of a crude method in designating patients into the AgP or CP group. Obviously one can argue why we should concern ourselves for a complicated method while by taking the age of 35 as a cutoff point, one has a classification [38]. However we demonstrated that even in “noisy” samples (some AgP cases are declared CP and vice versa) a host immune classifier can work with arbitrary accuracy. We assume this kind of noise is present even with the established criteria for AgP diagnosis [5]. Recently a study using a transcriptome classifier with four supervised learning methods reported good prediction results by one of the four; AgP or CP diagnosis was strictly made by the established criteria [39]. The authors suggested possible heterogeneity within the AgP and CP classes based on the variability of the results of the four methods. The combined use of unsupervised with supervised learning methods can be an attempt to reduce true misclassification error [28]. We realize that the AgP diagnostic criteria for the patients of sample-2 limit the generalization ability of the ANN results based on it.

In conclusion, we demonstrated by KDE methods possible evidence of two clusters on clinical and most immunological data from periodontitis patients. By the use of ANNs we can effectively classify periodontitis patients by their immune response profile into the AgP or CP class. We anticipate that future work on bigger samples extending the results of the present study and employing a wider array of parameters can turn personalized treatment of periodontitis from concept to reality.

## Supporting Information

File S1 **This file includes the following:** **Text S1.** Selection of a kernel function; Fitting clinical datasets to probability models; Constructing multilayer perceptron neural networks. **Table S1.** The fit of probability distribution models to clinical and immunological data. **Table S2.** Input feature selection by automatic relevance determination. **Table S3.** Comparison of two multivariate analyses to artificial neural networks.
(DOC)

Figure S1 **An example of quantile to quantile plots.**
(TIFF)
