# Supplementary material for: Artificial Neural Networks for the Diagnosis of Aggressive Periodontitis Trained by Immunologic Parameters
Source: PLoS One. 2014 Mar 6;9(3):e89757. doi: 10.1371/journal.pone.0089757 (PMC3945718; doi:10.1371/journal.pone.0089757)
Supplement: File S1 — This file includes the following: Text S1. Selection of a kernel function; Fitting clinical datasets to probability models; Constructing multilayer perceptron neural networks. Table S1. The fit of probability distribution models to clinical and immunological data. Table S2. Input feature selection by automatic relevance determination. Table S3. Comparison of two multivariate analyses to artificial neural networks. (DOC) [file pone.0089757.s001.doc]

**Supporting Information**

**Text S1**

**Selection of a kernel function**

Given a sample of *n* observations *X_1_, X_2_…X_n_* we define the density estimator [9] as

$\hat{f}(x;h)=\frac{1}{nh}\sum_{i=1}^{n} K(\frac{x-X_{i}}{h})$ (A.1),

where *h* is the bandwidth or smoothing parameter and *K* a kernel function. The choice of K and *h* determines the amount of smoothing of the data [18]. The choice of *K* is less important than the choice of *h*. We would like to have a value of *h* that minimizes the distance between the real and the estimated density. The optimal selection of *h* is the one that minimizes the mean integrated squared error (*MISE*) [10] given by the expression

$MISE(\mathop{f}\limits^{\wedge} (x))=E\int\{\mathop{f}\limits^{\wedge} (x;h)-f(x)\}^{2}dx$ (A.2),

with *E* standing for expected.

To estimate *h* we follow Silverman’s rule-of-thumb formula which is

$h=\sigma^{\wedge} C_{v}(k)n^{-1/(2v+1)}$ (A.3),

where $\sigma^{\wedge}$is the standard deviation of the mean of the sample, *v* the order of the kernel and *C_v_*(*k*) a constant of proportionality specific for the chosen kernel. The order *v* of a kernel is the first non-zero moment of it.

For this study, we select the Epanechnikov kernel [18] given by the expression

$K(u)=\left\{\begin{matrix} \frac{3}{4}(1-\frac{1}{5}u^{2})/\sqrt{5} if\left|u\right|<\sqrt{5} \\ 0 otherwise \end{matrix}\right.$ (A.4).

Since *K(u)* is non-negative and integrates to 1, it can serve as a probability density function (pdf). As a symmetric pdf it is also of second order (*v* = 2). Thus, for the Epanechnikov kernel the constant in (A.3) is estimated as *C_v_*(*k*) = 2.34 for *v* = 2. The Epanechnikov kernel is often called the optimal kernel, since with optimal bandwidth it yields the lowest *MISE* [10].

For multivariate joint density estimation the density estimator (A.1) becomes

$\mathop{f}\limits^{\wedge} (x)=\frac{1}{nh_{1}h_{2}....h_{D}}\sum_{i=1}^{n} K(\frac{x-X_{i}}{h_{1}})K(\frac{x-X_{i}}{h_{2}})....K(\frac{x-X_{i}}{h_{D}})$ (A.5)

and the rule-of-thumb bandwidth *h_D_* gives

$h_{D}=\mathop{\sigma_{D}}\limits^{\wedge} C_{v}(k,q)n^{-1/(2v+q)}$ (A.6)

with *q* is the number of variables. For the Epanechnikov kernel and for *q* = 2 and *v* = 2 we have *C_v_*(*k,q*) = 2.20.

Since we are interested in bivariate joint KDE, instead of taking the product of two univariate Epanechnikov kernels as in equation (A.5) we opted for the bivariate Epanechnikov kernel given by the expression

$K(u_{1},u_{2})=\left\{\begin{matrix} (2\pi)^{-1}(1-u_{1}^{2}-u_{2}^{2}) if\text{ }u_{1}^{2}+u_{2}^{2}<1, \\ 0 otherwise\text{ } \end{matrix}\right.\text{ } \text{ }$ (A.7).

The reason for the above choice is that Formula (A.5) assumes some kind of independence between the variables *X_i_*, while (A.7) does not. Therefore, the bivariate Epanechnikov kernel assigns equal weight to two points that are equidistant from the reference point.

When a dataset was found to have a bounded support as for instance over an interval (*α*, *β*), we needed to transform the data in values extending over the full interval (-∞, +∞) [9]. To this end we employ the transformation

$g(x)=\log\left(\frac{x-\alpha}{\beta-x}\right)$ (A.8),

and the kernel function assumes the form

$\mathop{t}\limits^{\wedge} (x)=\mathop{f}\limits^{\wedge} (\log(\frac{x-\alpha}{\beta-x})) (\frac{1}{x-\alpha}+\frac{1}{\beta-x})$ (A.9).

**Fitting clinical datasets to probability models**

A parametric approach assumes a known distribution model, while the objective of fitting distribution models to data is to find the most appropriate type of model and parameter values (mean, variance, etc) that give the highest probability of producing the observed data. Investigating the fit of clinical and immunologic data to probability distribution models, we first used Q-Q plots to visually identify competing candidate models (an example is shown in Figure S1), which we subsequently evaluated by their *AIC* scores [22] (Table S1).

The so–called Akaike information criterion (*AIC*) [20] is given by the following expression

$AIC=(\frac{n-2k+2}{n-k+1})-2\ln(L_{\max})$ (B.1),

where *n* is the number of observations in a dataset, *k* the number of parameters involved in the probability density function (pdf) of the model and *L_max_* the maximum joint probability density for the observed data defined, for instance for *k* = 2, as

$L_{\max}(x,\lambda^{\wedge} ,\theta^{\wedge} )=\prod_{i} f(x_{i},\lambda^{\wedge} ,\theta^{\wedge} )$ (B.2),

where $\lambda^{\wedge}$, $\theta^{\wedge}$ are the maximum likelihood estimation (*MLE*) values of these parameters and *f*(*x,λ,θ*) the corresponding pdf of the model under consideration [21].

In general, the model with the least *AIC* value is promoted. However, the differences by *AIC* between two models were tested for statistical significance using the *χ^2^* test based on the following expression [22]

$AIC_{i}-AIC_{i+j}+2=\chi_{j}^{2}$ (B.3),

where *i* is the degrees of freedom of the first model (number of parameters involved in the pdf of the model) and *j* the difference in the degrees of freedom between the two models.

The pdfs of the five models under consideration are given by the well known formulas [19]:

The Exponential model$f(x)=\lambda\exp(-\lambda x)$ for x ≥ 0, (otherwise *f(x)* = 0) (B.4),

The Weibull model $f(x)=\frac{\theta}{\lambda}(\frac{x}{\lambda})^{\theta-1}\exp-(x/\lambda)^{\theta}$for x ≥ 0, (otherwise *f(x)* = 0) (B.5),

The Pareto model $f(x)=\frac{\theta x^{\theta}_{\min}}{x^{\theta+1}}$ for *x* ≥ *x*_min_ with *x*_min_ = *λ* (B.6),

The Gamma model $f(x)=\frac{x^{\theta-1}\exp(-x/\lambda)}{\Gamma(\theta)\lambda^{\theta}}$ with $\Gamma(\theta)=(\theta-1)!$ (B.7),

where *λ* is a scale factor and *θ* is a shape factor in the above functions.

Finally the Normal model is given by $f(x)=\frac{1}{\sqrt{2\pi\sigma^{2}}}\exp(-\frac{(x-\mu)^{2}}{2\sigma^{2}})$ (B.8), with *μ* the mean and *σ*^2^ the variance of the sample’s distribution.

To derive a value for *L_max_* from equation B.2 we need the *MLE* estimates$\lambda^{\wedge}$and$\theta^{\wedge}$*.* Estimating $\lambda^{\wedge}$and $\theta^{\wedge}$by the *MLE* method is not always an easy task. Among various techniques we may try to solve the equation

$\frac{\partial L(x\left|\lambda\right.)}{\partial\lambda}=0$ (B.9).

Often, but not always, $\lambda^{\wedge}$ is a solution of (B.9). Nevertheless, from the literature [19], we may use the formulas,

$\lambda^{\wedge} =n/\sum_{i=1}^{n} (x_{i}-x_{\min})$, for pdf (B.4), while for equation (B.5) we know that $\theta^{\wedge}$ can be obtained iteratively from $\sum_{i=1}^{n} (x_{i}^{\theta}\ln x_{i})/\sum_{i=1}^{n} x_{i}^{\theta}-\theta^{-1}-\frac{1}{n}\sum_{i=1}^{n} \ln x_{i}=0$ to yield $\lambda^{\wedge} =\frac{1}{n}\sum_{i=1}^{n} x_{i}^{\theta^{\wedge} }$. For pdf (B.6) we have $\lambda^{\wedge} =x_{i\min}$ and $\theta^{\wedge} =\frac{n}{\sum_{i=1}^{n} \log(x_{i}/\lambda^{\wedge} )}$, for (B.7) $\theta^{\wedge}$ is determined iteratively from , where is the digamma function, yielding$\lambda^{\wedge} =\frac{1}{\theta^{\wedge} n}\sum_{i=1}^{n} x_{i}$ and for (B.8) we have $\mathop{\mu^{\wedge} =\frac{1}{n}\sum_{i=1}^{n} x_{i}}\limits$and $\mathop{\sigma^{2}}\limits^{\wedge} =\frac{1}{n}\sum_{i=1}^{n} (x_{i}-\mu^{\wedge} )^{2}$.

The Cumulative Probability Functions (cpfs) are given by the following formulas [19]:

For the Exponential model: $F(x;\lambda)=1-\exp(-\lambda x)$for *x*≥0 (0 if *x*<0) (B.10),

For the Weibull model: $F(x;\lambda,\theta)=1-\exp[-(x/\lambda)^{\theta}]$ (B.11),

For the Pareto model: $F(x;x_{m},\theta)=1-(\frac{x_{m}}{x})^{\theta}$for *x*≥*x_m_*  (0 if *x*< *x_m_*) (B.12),

For the Gamma model: $F(x;\lambda,\theta)=\frac{\gamma(\theta;x/\lambda)}{\Gamma(\theta)}$ (B.13),

where *γ* is the lower incomplete gamma function.

For the Normal model: $F(x;\mu,\sigma^{2})=\frac{1}{2}[1+erf(\frac{x-\mu}{\sigma\sqrt{2}})]$ (B.14),

where *erf* is the Error function.

**Constructing multilayer perceptron neural networks**

At the hidden layer the network computes the activation of *CE* values by summing all values using arbitrary weights (*w_i_*) to initiate the network as

$A=\sum_{t=1}^{N} w_{i}CE_{i}$ (C.1).

The sum is transferred to the outcome layer by the sigmoid logistic function, given by

$Outcome=\frac{1}{1+\exp(-A)}$ (C.2),

where the final result of the network exits as AgP or CP class through again the sigmoid logistic function [12]. Function (C.2) gives 1 (if outcome is close to 1) or 0 (if outcome is close to 0).

Synaptic weights in the network are adjusted to the classification error of the outcome produced by the network itself. Positive weights in the synapses of the network are considered excitatory and negative ones inhibitory. The error of the classification is estimated as

$E=\frac{1}{2}\sum_{i=1}^{N} (t-y_{i})^{2}$ (C.3),

where *t* is the target (desired) outcome set to a “reachable” value 0.9 or 0.1 by the network (and not 1 or 0) and *y* the outcome of the network.

The error signal (C.3) propagates backwards and trains the ANN by iteratively reducing weights (*w_i_*) by a factor

$\Delta_{new}=-\eta\frac{\partial E}{\partial w_{i}}$ (C.4).

*η* is an arbitrarily chosen number from the domain [0,1] called the learning rate. To regularize weight decay we can apply a penalty to the error function (C.3) and then C.4 becomes

$\Delta_{new}=-\eta\frac{\partial E}{\partial w_{i}}-\eta\lambda w_{i}$ (C.5)

where *λ* is a regularization parameter. The old weights in the iterative procedure described above are multiplied by a momentum coefficient (*m*) from the domain [0,1] which reduces rapid fluctuations and aids to convergence.

Using (C.1) and (C.2) with *y = outcome* and *CE_i_ = x_i_* and differentiating (C.3) with respect to *w_i_* we obtain the backpropagation logarithm called gradient descent

$\frac{\partial E}{\partial w_{i}}=\frac{\partial E}{\partial y_{i}}\frac{\partial y_{i}}{\partial w_{i}}=(y_{i}-t)y(1-y_{i})x_{i}$ (C.6).

By the iterative procedure described above (each iteration is called epoch) we perform supervised learning till we minimize the error of the network to the maximum extent. A software package (SPSS v 17.0 programme, IBM, Chicago) was used for running the networks.

10-fold cross validation for the determination of the weight decay regularization parameter *λ* entailed random partitioning of the sample to 10 equally sized groups. We trained the subsample of the 9-groups with the classifier and estimated the error of the network on the “left out” 10th group. In training the 9-groups subsamples we activated the automatic relevance determination for input feature selection for each step of the cross validation. In this way input features were not fixed for all steps and the “left out” group remained unknown to the training process. We iterated the processes until all subgroups were used as “left out” group and we aggregated the error. We repeated the process for 12 values in the range from 0.1 to 0.00005 and selected the value producing the least 10-fold validation score.

Cases with missing values were excluded from the ANNs analysis. While there were very few missing values in samples 2, 3 and sample-4 for IgG titers, they were quite apparent in data for interleukins (sample-4). The size of the sample in the latter case was reduced by 30%.

| Table S1. The fit of probability distribution models to clinical and immunological data from previous studies.^a,b,c,d,e^ The promoted model by the least Akaike information criterion (*AIC*) value is highlighted in bold. Symbol *λ* is a scale and *θ* a shape parameter in the models’ probability density functions (pdfs), computed by the maximum likelihood estimation (*MLE*) method. Symbol σ is the standard deviation and $\bar{x}$the mean value for the normal model. | | | | |
| --- | --- | --- | --- | --- |
| Parameter | Competing models | *MLE* estimation  of pdf parameters | *AIC*-value | p-value  by the *x^2^* test |
| Radiographic bone loss level^a^ | | | | |
| Baseline  mean radiographic bone loss level | Gamma (G)  vs  **Normal (N)** | G(*λ*) = 0.292  G(*θ*) = 24.191  **N** (*σ*) = 1.439  **N**$(\bar{x})$= 7.079 | G = 216.60  **N** = 103.38 | <10^-5^ |
| Longitudinal mean radiographic bone loss level change | Gamma (G)  vs  **Normal (N)** | G (*λ*) = 0.272  G (*θ*) = 1.093  **N** (*σ*) = 0.285  **N**$(\bar{x})$= 0.298 | G = 28.02  **N** = 9.42 | <10^-5^ |
| Lymphocytes^b^ | | | | |
| Total lymphocytes | Gamma (G)  vs  **Normal (N)** | G (*λ*) = 0.153  G (*θ*) = 12.852  **N** (*σ*) = 0.547  **N**$(\bar{x})$= 1.961 | G = 149.51  **N** = 131.99 | <10^-5^ |
| CD4/CD8^f^ ratio | **Gamma (G)**  vs  Weibull (W) | **G** (*λ*) = 0.342  **G** (*θ*) = 5.904  W (*λ*) = 1.091  W (*θ*) = 0.137 | **G** = 174.97  W = 550.37 | <10^-5^ |
| CD3 | **Gamma (G)**  vs  Exponential (E) | **G** (*λ*) = 0.128  **G** (*θ*) = 11.390  E (*λ*) = 1.162 | **G** = 85.10  E = 236.14 | <10^-5^ |
| CD19^g^ | **Weibull (W)** | **W** (*λ*) = 0.704  **W** (*θ*) = 0.266 |  |  |
| Leukocytes^c^ | | | | |
| Monocytes | **Gamma (G)**  vs  Normal (N) | **G** (*λ*) = 0.048  **G** (*θ*) = 9.204  N (*σ*) = 0.146  N$(\bar{x})$= 0.444 | **G** = -78.27  N = -73.47 | 0.009 |
| Eosinophiles | **Gamma (G)**  vs  Normal (N) | **G** (*λ*) = 0.051  **G** (*θ*) = 3.109  N (*σ*) = 0.089  N$(\bar{x})$= 0.158 | **G** = -144.79  N = -143.34 | 0.063 (NS) |
| Neutrophils | **Gamma (G)**  vs  Weibull (W) | **G** (*λ*) = 0.661  **G** (*θ*) = 5.776  W (*λ*) = 10.290  W (*θ*) = 1.672 | **G** = 265.43  W = 402.49 | <10^-5^ |
| Basophils^g^ | **Normal (N)** | **N** (*σ*) = 0.025  **N**$(\bar{x})$= 0.056 |  |  |
| Immunoglobulins^d^ | | | | |
| IgG^h^ | **Gamma (G)**  vs  Normal (N) | **G** (*λ*) = 0.584  **G** (*θ*) = 17.398  N (*σ*) = 2.434  N$(\bar{x})$= 10.153 | **G** = 362.65  N = 369.36 | 0.0032 |
| IgA | **Gamma (G)**  vs  Weibull (W) | **G** (*λ*) = 0.419  **G** (*θ*) = 5.049  W (*λ*) = 1.289  W (*θ*) = 0.369 | **G** = 205.65  W = 428.54 | <10^-5^ |
| IgM | **Gamma (G)**  vs  Weibull (W) | **G** (*λ*) = 0.539  **G** (*θ*) = 3.024  W (*λ*) = 1.172  W (*θ*) = 0.389 | **G** = 185.07  W = 372.79 | <10^-5^ |
| Host response molecules^e^ | | | | |
| IL-1^i^ | Gamma (G)  vs  **Weibull (W)** | G (*λ*) = 418.58  G (*θ*) = 0.534  **W** (*λ*) = 233.09  **W** (*θ*) = 1.006 | G = 583.02  **W** = 578.52 | 0.011 |
| IL-2 | **Gamma (G)**  vs  Weibull (W) | **G** (*λ*) = 4.196  **G** (*θ*) = 1.082  W (*λ*) = 1.251  W (*θ*) = 0.168 | **G** = 284.34  W = 469.18 | <10^-5^ |
| IL-4 | **Gamma (G)**  vs  Exponential (E) | **G** (*λ*) = 4.876  **G** (*θ*) = 2.235  E (*λ*) = 0.092 | **G** = 294.44  E = 312.74 | <10^-5^ |
| IL-6 | **Gamma (G)**  vs  Weibull (W) | **G** (*λ*) = 374.55  **G** (*θ*) = 0.550  W (*λ*) = 17.11  W (*θ*) = 0.564 | **G** = 458.33  W = 549.77 | <10^-5^ |
| TNF-α^j^ | Gamma (G)  vs  **Exponential (E)** | G (*λ*) = 359.94  G (*θ*) = 1.322  **E** (*λ*) = 0.002 | G = 843.66  **E** = 816.96 | <10^-5^ |
| INF-γ^k^ | **Gamma (G)**  vs  Weibull (W) | **G** (*λ*) = 96.016  **G** (*θ*) = 0.541  W (*λ*) = 1.229  W (*θ*) = 0.063 | **G** = 539.26  W = 757.87 | <10^-5^ |
| IgG antibody titers | | | | |
| *A.a*.^l^ titers | Gamma (G)  vs  **Exponential (E)** | G (*λ*) = 12.229  G (*θ*) = 0.132  **E** (*λ*) = 0.493 | G = 453.14  **E** = 398.11 | <10^-5^ |
| *P.g*.^m^ titers | Gamma (G)  vs  **Exponential (E)** | G (*λ*) = 26.205  G (*θ*) = 0.273  **E** (*λ*) = 0.131 | G = 846.57  **E** = 839.60 | 0.0027 |
| *C.o*.^n^ titers | Normal (N)  vs  **Exponential (E)** | N (*σ*) = -0.073  N$(\bar{x})$= 0.607  **E** (*λ*) = 0.901 | N = -8.08  **E** = -9.09 | 0.083 (NS) |
| ^a^ (Papantonopoulos, 2004)  ^b^ (Loos *et al*., 2004)  ^c^ (Loos *et al*., 2000)  ^d^ (Graswinckel *et al*., 2004)  ^e^ (Takahashi *et al*., 2001)  ^f^ CD = cluster of differentiation  ^g^ no second competing model was indicated by Q-Q plots  ^h^ Ig = immunoglobulin  ^i^ IL= interleukin  ^j^ TNF-α = tumor necrosis factor  ^k^ INF-γ = interferon  ^l^ *A.a*. = *Aggregatibacter actinomycetemcomitans*(Y4 antigen)  ^m^ *P.g*. = *Porphyromonas gingivalis*(FDC381 antigen)  ^n^ *C.o. = Capnocytophaga ochracea* | | | | |

| Table S2. Input feature selection by automatic relevance determination (ARD). Features showing low posterior weight distribution variance are excluded (in bold the selected features). These results are generated using the whole samples from which parameters originate. During 10-fold cross validation for determining the weight decay regularization parameter of neural networks, ARD is repeated at each step, but each time with a “left out” subgroup not included. | |
| --- | --- |
| Parameter | Posterior weight distribution variance |
| ANN_1_ | |
| **CD4/CD8^a^**  CD3  CD19  Lymphocytes  **Neutrophils**  **Monocytes**  **Eosinophils**  Basophils | **3.026**  0.592  1.340  1.663  **2.477**  **2.438**  **2.999**  0.000 |
| ANN_2_ | |
| **IL-1^b^**  IL-2  **IL-4**  IL-6  **TNF-α^c^**  **IFN-γ^d^** | **10.363**  5.537  **12.425**  5.459  **9.985**  **18.678** |
| ANN_3_ | |
| ***A.a.*(Y4) titers^e^**  *A.a.* (ATCC 29523) titers  *A.a.* (SUNY67) titers  ***C.o*. (S3) titers ^f^**  *E.c*. (ATCC 23834) titers ^g^  ***F.n*. (ATCC 25586) titers ^h^**  *P.i.* (ATCC 25611) titers ^i^  *P.n.* (ATCC 33563) titers ^k^  ***P.g.*** **(FDC381) titers ^l^**  *P.g.* (SU63) titers  *T.d.* (ATCC 35405) titers ^m^  *W.s.* (ATCC 29543) titers ^n^ | **11.207**  0.757  1.727  **4.666**  0.829  **4.269**  3.032  2.298  **5.019**  3.076  0.359  3.296 |

^a^ CD = cluster of differentiation

^b^ IL= interleukin

^c^ TNF-α = tumor necrosis factor

^d^ INF-γ = interferon

^e^ *A.a*. = *Aggregatibacter actinomycetemcomitans*

^f^ *C.o. = Capnocytophaga ochracea*

^g^ *E.c. = Eikenella corrodens*

^h^ *F.n. = Fusobacterium nucleatum*

^i^ *P.i. = Prevotella intermedia*

^k^ *P.n. = Prevotella nigrescens*

^l^ *P.g. = Porphyromonas gingivalis*

^m^ *T.d.* = *Treponema denticola*

^n^ *W.s. =Wolinella succinogens*

| Table S3. Two multivariate analyses that produce inferior results compared to artificial neural networks (ANNs) reported in Table 1. Input features are the same as with ANNs selected for them (in bold) by automatic relevance determination (ARD). | | |
| --- | --- | --- |
| Input features | Canonical discriminant analysis  Sens. Spec. | Binary logistic regression  Sens. Spec. |
| ANN_1_ | | |
| **CD4/CD8^a^**  CD3  CD19  Lymphocytes  **Neutrophils**  **Monocytes**  **Eosinophils**  Basophils | All features  62.5% 72.3%  selected by ARD  50.0% 72.3% | All features  55.6% 71.9%  selected by ARD  50.0% 72.3% |
| ANN_2_ | | |
| **IL-1^b^**  IL-2  **IL-4**  IL-6  **TNF-α^c^**  **IFN-γ^d^** | All features  75.0% 75.0%  selected by ARD  80.0% 77.5% | All features  75.0% 80.6%  selected by ARD  80.0% 77.5% |
| ANN_3_ | | |
| IgG titers of:  ***A.a.*(Y4) ^e^**  *A.a.* (ATCC 29523)  *C.r*. (ATCC 33238) **^f^**  ***C.o*. (S3) ^g^**  *E.c*. (ATCC 23834) ^h^  ***F.n*. (ATCC 25586) ^i^**  *P.i.* (ATCC 25611) ^k^  *P.n.* (ATCC 33563) ^l^  ***P.g.* (FDC381) ^m^**  *P.g.* (SU63)  *T.d.* (ATCC 35405) ^n^  *W.s.* (ATCC 29543) ^o^ | All features  71.1% 66.7%  selected by ARD  49.1% 75.0% | All features  73.8% 78.3%  selected by ARD  50.0% 64.2% |

^a^ CD = cluster of differentiation

^b^ IL= interleukin

^c^ TNF-α = tumor necrosis factor

^d^ INF-γ = interferon

^e^ *A.a*. = *Aggregatibacter actinomycetemcomitans*

^f^ *C.r. = Campylobacter rectus*

^g^ *C.o. = Capnocytophaga ochracea*

^h^ *E.c. = Eikenella corrodens*

^i^ *F.n. = Fusobacterium nucleatum*

^k^ *P.i. = Prevotella intermedia*

^l^ *P.n. = Prevotella nigrescens*

^m^ *P.g. = Porphyromonas gingivalis*

^n^ *T.d.* = *Treponema Denticola*

^o^ *W.s. =Wolinella succinogens*

**Figure S1. legend**

Quantile to quantile plots (Q-Q plots): The fit on the diagonal line indicates how close the tested distribution approximates a probability model. This can serve as the first visual inspection for a subsequent analysis by information-theoretic criteria. Here four Q-Q plots for the monocytes dataset.
