# Supplementary figures and images for: Artificial Neural Networks for the Diagnosis of Aggressive Periodontitis Trained by Immunologic Parameters
Source: PLoS One. 2014 Mar 6;9(3):e89757. doi: 10.1371/journal.pone.0089757 (PMC3945718; doi:10.1371/journal.pone.0089757)

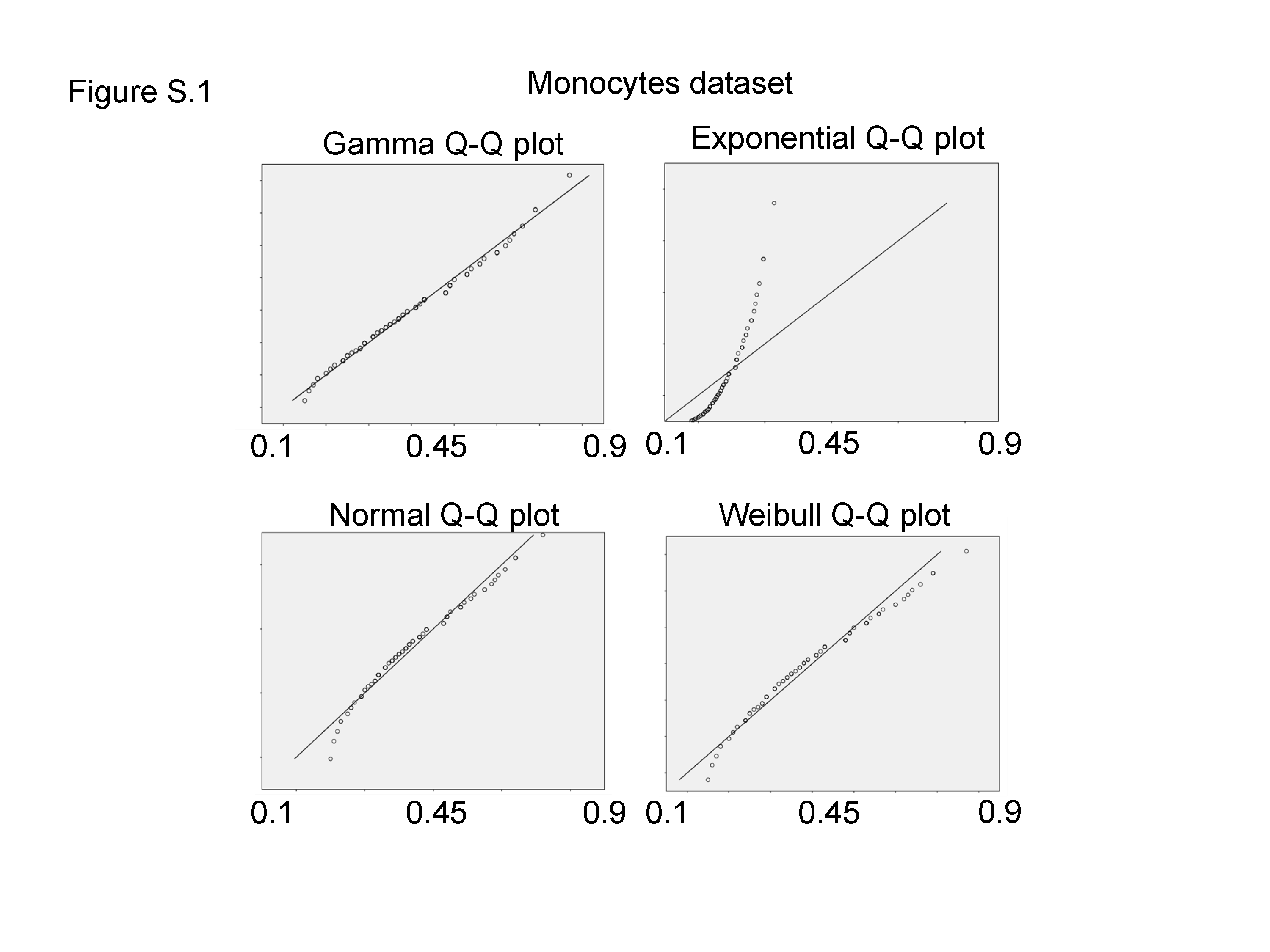

Supplement: Figure S1 — An example of quantile to quantile plots. (TIFF) [file pone.0089757.s002.tif]
